# A Breathable, Highly Sensitive, and Wearable Piezoresistive Sensor with a Wide Detection Range Based on Gradient Porous PU@MXene/CNT Film for Electronic Skin

**DOI:** 10.3390/polym17111530

**Published:** 2025-05-30

**Authors:** Xiuli Yang, Feiran He, Huihui Qiao, Shuibo Yang, Dehua Wen, Kaige Yang, Ziyi Dang, Yin He

**Affiliations:** 1 School of Textile Science and Engineering, Tiangong University, Tianjin 300387, China; 13011335551@126.com (X.Y.); 15064768326@163.com (F.H.); 18222477692@163.com (H.Q.); 14777537638@163.com (S.Y.); 13211079562@163.com (D.W.); yangkaige2022@163.com (K.Y.); 13772715657@163.com (Z.D.); 2 Shaoxing Keqiao Institute, Tiangong University, Shaoxing 312030, China; 3 Institute of Intelligent Wearable Electronic Textiles, Tiangong University, Tianjin 300387, China; 4 Ministry of Education Key Laboratory for Advanced Textile Composite Materials, Tiangong University, Tianjin 300387, China

**Keywords:** piezoresistive sensor, gradient porous film, electronic skin

## Abstract

Developing flexible sensors that combine high sensitivity, a wide detection range, and comfortable wearability remains a key challenge in the development of electronic skin. This study presents a breathable, highly sensitive, and wearable piezoresistive sensor based on the preparation of hierarchical microporous PU@MXene + CNT films and single-sided electrodes using a simple and effective method. Distilled water was used as a non-solvent to induce the separation of polyurethane films (PU) with different mass fractions, forming a gradient porous structure with inconsistent pore morphologies in the upper and lower layers. Three-dimensional structure analysis of the hierarchical porous films with varying gradients, conducted using computed tomography, revealed that the porous structures formed after phase separation of PU solutions with different mass fractions exhibited different morphologies. As the mass fraction increased, the pore size, pore volume, and porosity gradually decreased while the surface area gradually increased. The greater the gradient of the constructed porous film, the more significant the difference between the upper- and lower-layer structures. A flexible sensor prepared using the PU@MXene + CNT porous film with the largest gradient exhibited excellent sensitivity in a wide detection range from 0.7 to 20 kPa, which was higher than that of porous films with other gradients, demonstrating high stability (>8000 cycles). The air permeability and moisture permeability of PU@MXene + CNT with the largest gradient were 0.9922 L/m^2^/s and 1123.6 g/m^2^/day, respectively, and these values were 1.35 and 4.40 times those of the non-porous film. Therefore, the constructed flexible piezoresistive sensor with a gradient porous structure had both high sensitivity and wide detection range, as well as good air and moisture permeability. Finally, the sensor successfully monitored human movements, including throat activity, finger motions, and arm bending, demonstrating its potential for wearable electronic applications.

## 1. Introduction

Wearable, flexible sensors have attracted significant attention owing to their potential applications in healthcare, human–machine interaction, and motion monitoring [1,2,3,4,5,6]. Wearable piezoresistive force-sensitive sensors have a simple sensing mechanism, converting external pressure stimuli into electrical signals or other signal outputs, which serve as an important component of wearable, flexible sensors [7,8,9]. However, most reported skin-type pressure sensors lack breathability, leading to skin discomfort and inflammation during prolonged use [10,11]. Therefore, wearable sensors with breathable and sensitive piezoresistive materials are needed for health monitoring [12,13].

Many microstructures have been used to improve the sensitivity of force-sensitive sensors [14], including pyramids [10], irregular [11], sandpaper-like [15], and honeycomb structures [16]. Compared to other types of force-sensitive sensors, force-sensitive sensors with surface microstructures typically have a lower detection limit, as well as lower sensitivity with high pressure. Therefore, a key challenge is increasing conductive paths under high pressure to maintain sensitivity across a wide pressure range. Specifically, porous structures offer advantages over other microstructures in terms of sensitivity and detection range. Unlike sensors based on other microstructures, with increasing pressure, the sensitivity of porous structures remains limited by the rapid saturation of the surface conductive paths under pre-stress accumulation. Due to the porous structure, applying external pressure causes the pores to first decrease in size, causing the pore walls to come into contact and causing the pore walls to be further squeezed. This process can continuously cause changes in the conductive paths [17,18]. This dynamic response enables high sensitivity across a wide pressure range, addressing the industry challenge of simultaneously achieving high sensitivity and a broad detection limit.

Porous conductive polymer composites (CPCs) exhibit excellent flexibility and sensitivity, making them well-suited for use as piezoresistive materials [19,20,21,22,23]. Their three-dimensional porous structure enhances performance by reducing the percolation threshold of the conductive filler, improving sensitivity, and optimizing weight and compressibility [21]. For example, Huang et al. prepared a carbon nanotube (CNT)/thermoplastic polyurethane (TPU) oriented conductive foam with a percolation threshold of 0.0023 vol%, which improved its mechanical properties [22]. Under a strain of 90%, the stress of the aligned foam was 22 times higher than that of disordered foam. Huang et al. successfully prepared sponges containing CNTs, graphene, cellulose nanofibers/silver nanowires, and PU [24,25,26]. In addition, a carbon nanotube-polydimethylsiloxane (CNT-PDMS) porous composite prepared by Han et al. [27] exhibited a pressure response range of 0–110 kPa and a high sensitivity of 5.6 kPa^−1^.

Although porous CPCs have excellent deformation capabilities, their uniform pore structure can lead to large internal energy dissipation of the elastic material, resulting in a significant decrease in the sensitivity of the sensor under a large pressure range. Shen et al. [28] prepared a plant fiber porous sponge and found that the sensitivity decreased from 133.3 kPa^−1^ in the low-pressure range (0.01–0.75 kPa) to 1.89 kPa^−1^ under high pressure (0.75–10 kPa). To increase the sensitivity of the sensor under high pressure, Zhao et al. adopted a non-uniform ratio configuration with upper and lower layers, which was used to construct a gradient porous structure with upper and lower layers [29]. The prepared, flexible sensor still exhibited high sensitivity under high pressure. The results showed that designing a hierarchical gradient porous structure could effectively improve the sensitivity of the flexible sensor under high pressure and further enhance the practical performance of the sensor.

CPCs with a porous structure are permeable to air and moisture. Sponges, foams, and aerogels prepared using methods such as sacrificial templating, freeze-drying, and foaming serve as common materials for porous CPCs [19,30,31,32]. However, these preparation methods produce materials that are too thick, exceeding 5 mm [3,32,33,34], making them unsuitable for fabricating ultra-thin, skin-like sensors. In addition, few studies have investigated the application of prepared porous-structured polymer films thinner than 1 mm in high-performance piezoresistive sensors.

Building on the above, this study proposed a breathable, high-performance, and long-term wearable piezoresistive sensor based on a gradient porous CPC film. The fabrication process combined non-solvent (distilled water) induced phase separation (NIPS) technology with traditional solution casting. Polyurethane (PU) solutions with different mass fractions were selected for the upper and lower layers to rapidly form a hierarchical structure with inconsistent upper and lower layers, which was solidified into a gradient porous film. This preparation method was simple, controllable, and energy efficient. During the preparation process, PU films with different gradient pore structures were prepared by controlling the PU concentration. MXenes and CNTs were attached to the pores of the PU porous film through dip-coating to form a uniform conductive network. Three-dimensional imaging analysis of the PU@MXene + CNT porous film was carried out by micro-computed tomography (Micro-CT) technology to obtain the real internal structure, and a quantitative analysis of its structural parameters was conducted. The air holes in the film contained different volume distributions, and the upper and lower layers of the gradient porous structure had different pore morphologies. The larger the mass fraction of PU, the smaller the formed pore volume and pore size, and the lower the porosity. In addition, the greater the difference in mass fraction between the two layers, the higher the gradient degree. This work studied the effects of pore volume distribution, porosity, electrical properties, tensile resistance, air permeability, and moisture permeability of the gradient porous films with different parameters, as well as their impacts on sensor performance. The results showed that a highly graded porous structure provided the best overall performance, maintaining high sensitivity across a wide pressure range while minimizing hysteresis and improving stability. In addition, a PU@MXene + CNT piezoresistive sensor was successfully used for human motion detection, capturing throat movements during swallowing, finger movements during pressing and bending, and arm bending motions.

## 2. Materials and Methods

### 2.1. Materials

PU (HK-3050, with a mass fraction of 30%) was obtained from Shanghai Beilu Chemical Technology Co., Ltd. (Shanghai, China), while CNTs were purchased from Chengdu Organic Chemicals Co., Ltd., Chengdu, China. The outer diameter of the CNTs was 4–6 nm, with a length of 0.3–2 μm and purity of >98%. Multilayer MXenes (Ti_2_C_3_) were purchased from Beijing Beike New Material Technology Co., Ltd. (Beijing, China), where the size of the MXenes was 1–5 μm. N, N-dimethylformamide (DMF) was purchased from Tianjin Comin Chemical Reagent Co., Ltd., Tianjin, China. Water was distilled in the laboratory without further treatment.

### 2.2. Preparation Process of the Porous PU@MXene + CNT Film

The preparation process consisted of two main steps: NIPS and dip coating. First, DMF and PU solutions were prepared with PU mass fractions of 10, 12, 14, and 16% and stirred in a 40 °C water bath for 45 min to form stable and uniform solutions. The solutions were then poured into syringes and allowed to stand for 1 h to remove any air bubbles. The PU solutions with mass fractions of 12, 14, and 16% were injected into glass molds to form Layer 1. The molds were then placed in beakers filled with distilled water and maintained at 20 °C for 12 h. After removal, dust-free paper was used to absorb surface moisture. The 10% PU solution was then injected as Layer 2, while a single-layer sample containing only Layer 2 was prepared as a control. The molds were returned to the beakers filled with distilled water and maintained at 20 °C for another 12 h. The porous PU was then peeled from the molds and dried under natural conditions. This process resulted in the formation of single-layer 10% PU@MXene + CNT films and double-layer 10%/12% PU@MXene + CNT, 10%/14% PU@MXene + CNT, 10%/16% PU@MXene + CNT composite films. The MXene + CNT dispersion was prepared by ultrasonic treatment, with a CNT concentration of 0.1 mg/mL and an MXene concentration of 1 mg/mL. Then, the porous PU film was immersed in the dispersion and subjected to ultrasonic treatment for 2 h to uniformly adhere the MXenes and CNTs to the film. Finally, the porous PU@MXene + CNT film was dried in an oven at 50 °C for 2 h.

This flexible piezoresistive sensor encapsulated the PU@MXene + CNT film using a combination of self-adhesive, non-woven liners and screen-printed single-sided electrodes. The single-sided electrodes made good contact with the PU@MXene + CNT film, and the encapsulation process did not affect the use of the porous PU@MXene + CNT film.

### 2.3. Characterization

The structure of the PU@MXene + CNT porous film was observed using a scanning electron microscope (SEM) (Regulus 8100, Hitachi, Tokyo, Japan) and a three-dimensional X-ray microscope (Xradia 510 Versa, Zeiss, Hebron, KY, USA). Raman spectroscopy was performed with a 532 nm laser excitation, a resolution of 1 cm^−^^1^, and a range of 50~4000 cm^−^^1^. An X-ray photoelectron spectrometer (K-alpha, Thermofisher, Waltham, MA, USA) was used for the qualitative and quantitative analysis of the surface composition of the composite film. The air permeability was measured using a fully automatic air permeability tester (Model YG461D-II, Wenzhou Darong Textile Instrument Co., Ltd., Wenzhou, China). The mechanical tensile properties of the samples were detected using a universal tensile testing machine. The length of the sample was 40 mm, and the tensile speed was 100 mm/min. The moisture permeability of the fabric was determined using a fabric moisture permeability tester (Model YG(B)216-II, Wenzhou Darong Textile Instrument Co., Ltd., Wenzhou, China). The piezoresistive characteristics of the sensor were tested using a self-developed flexible sensing performance detection system and a digital dual-display multimeter (U34410A, Agilent, Santa Clara, CA, USA). Each sensor underwent a compression force cycle experiment ranging from 10 to 10,000 times. The load was 0~6.3 N, and the measurement data point was 10 mm/s.

## 3. Results and Discussion

**Figure 1:**
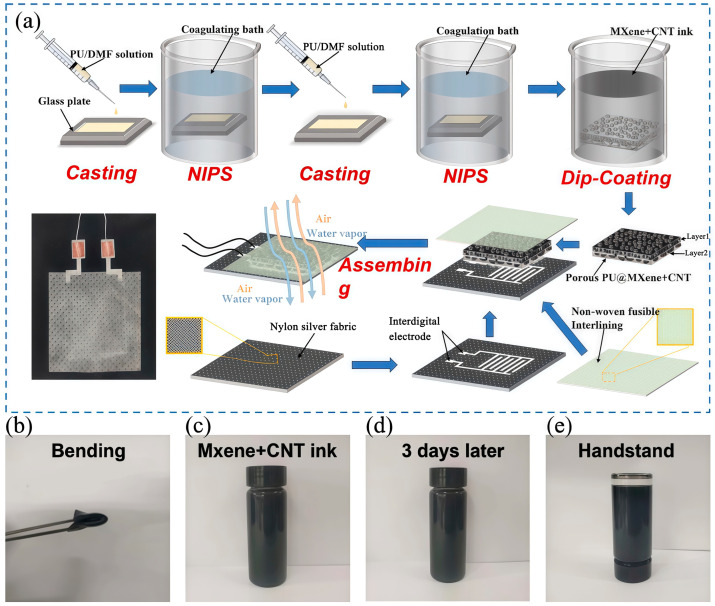
(**a**) Schematic diagram of the preparation process of the PU@MXene + CNT porous film and piezoresistive sensor; (**b**) photograph of the porous PU@MXene + CNT film; (**c**–**e**) images of the MXene + CNT dispersion liquid.

Figure 1a presents the manufacturing process of the gradient porous PU@MXene + CNT film and the breathable, wearable piezoresistive sensor. First, the gradient porous PU film was prepared by solution casting and NIPS methods, indicating that the porous morphology, such as the pore size, was affected by the PU mass fraction (10, 12, 14, and 16%). Layer 2 was prepared from a 10% PU solution, and Layer 1 was constructed with different PU solutions to form the gradient porous structure. During the mutual diffusion process between the solvent and the non-solvent, the solvent (DMF) migrates from the polyurethane (PU) solution into the deionized water, while the deionized water penetrates into the PU solution. This bidirectional diffusion triggers phase separation in the PU solution, leading to the formation of a polymer-rich phase and a solvent-rich phase. As the phase separation progresses, the solvent-rich phase gradually evolves into a porous structure, whereas the PU-rich phase solidifies to form the skeletal framework of the film, ultimately resulting in the construction of a porous-structured PU membrane. The MXenes and CNTs were applied to the prepared gradient porous PU film by impregnation. This preparation process of the gradient porous film offered various advantages, including simple operation, low cost, and the feasibility of large-scale production. The gradient porous PU@MXene + CNT film was then encapsulated using a self-adhesive non-woven liner, and a single-sided electrode screen-printed on a nylon-silver fabric was used to assemble the film into a wearable piezoresistive sensor. The self-adhesive, non-woven liner and nylon-silver fabric exhibited good permeability, which did not affect the sensor’s flexibility and permeability. The gradient porous PU@MXene + CNT sensor could permeate air and water vapor, as shown in Figure 1a, while Figure 1b presents a photograph of the fabricated gradient porous PU@MXene + CNT film, showing its excellent flexibility. Figure 1c presents the MXene + CNT mixed dispersion prepared by ultrasonication, while Figure 1d–e displays images of the dispersion after standing for 3 days and after it was inverted, indicating that no obvious precipitation occurred.

**Figure 2:**
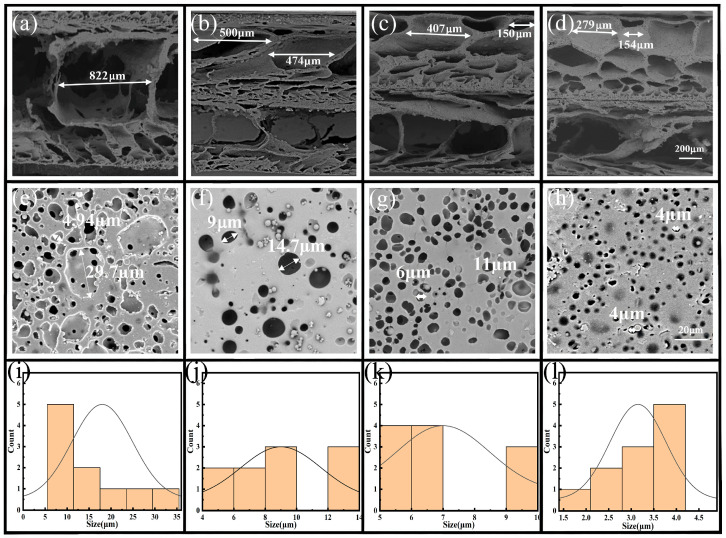
SEM images of the cross-sections of porous films with different gradients: (**a**) 10%PU@MXene + CNT (only Layer 2), (**b**) 10%/12%PU@MXene + CNT, (**c**) 10%/14%PU@MXene + CNT, (**d**) 10%/16%PU@MXene + CNT (Layer 1 on top and Layer 2 on bottom); SEM images of the surfaces of the porous films with different gradients: (**e**) 10%PU@MXene + CNT, (**f**) 10%/12%PU@MXene + CNT, (**g**) 10%/14%PU@MXene + CNT, (**h**) 10%/16%PU@MXene + CNT; surface pore size distribution states of the porous films with different gradients: (**i**) 10%PU@MXene + CNT, (**j**) 10%/12%PU@MXene + CNT, (**k**) 10%/14%PU@MXene + CNT, (**l**) 10%/16%PU@MXene + CNT.

Figure 2 presents the SEM images of the cross-sections and surfaces of the films with different gradients, as well as the pore size distribution diagrams of the surfaces. The images indicated that the pore morphology was affected by the PU concentration. Figure 2a–d shows the cross-sections of the films with different constructed pore gradients constructed. Among these, Layer 2 was formed by a 10% PU solution, and the pore morphology was nearly consistent. Layer 1 in Figure 2b–d was formed using 12, 14, and 16% PU solutions, where the pore size and pore volume decreased as the mass fraction increased. Figure 2e–h presents the surfaces formed by PU solutions with concentrations of 10, 12, 14, and 16%, indicating a notable decreasing trend in pore size. As shown in Figure 2i–l,10% of the films have a distribution range of 5~35 μm, 12% of the films have a distribution range of 4~14 μm, 14% of the films have a distribution range of 5~10 μm, and 16% of the films have a distribution range of 1.5~4.0 μm. The distribution range of the pore sizes on the surfaces of the gradient porous films gradually decreased as the mass fraction increased, indicating that the gradient porous structure referred to the obvious difference in pore morphology between the upper and lower layers. The pore morphology of the gradient porous films increased with increasing differences in concentration of the PU solutions used for the upper and lower layers. Specifically, the gradients of the four samples increased successively from left to right.

**Figure 3:**
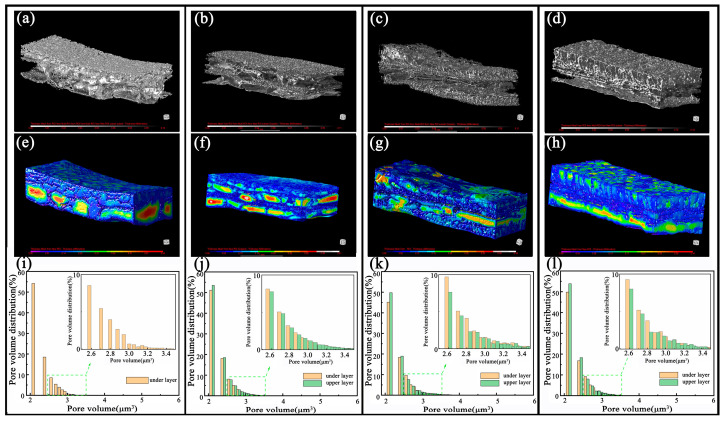
Micro-CT analysis diagrams of the structures of the porous films with different gradients: reconstructed diagrams of the PU skeletons of the films: (**a**) 10%PU@MXene + CNT, (**b**) 10%/12%PU@MXene + CNT, (**c**) 10%/14%PU@MXene + CNT, (**d**) 10%/16%PU@MXene + CNT; reconstructed diagrams of the pores of the films: (**e**) 10%PU@MXene + CNT, (**f**) 10%/12%PU@MXene + CNT, (**g**) 10%/14%PU@MXene + CNT, (**h**) 10%/16%PU@MXene + CNT; pore volume distribution diagrams of the films: (**i**) 10%PU@MXene + CNT, (**j**) 10%/12%PU@MXene + CNT, (**k**) 10%/14%PU@MXene + CNT, (**l**) 10%/16%PU@MXene + CNT.

The three-dimensional porous structures of the different PU@MXene + CNT films were analyzed using micro-computed tomography (micro-CT). Figure 3a–d presents the reconstructed diagrams of the PU skeletons of the films, and Figure 3e–h shows the reconstructed diagrams of the pores of the films. After micro-CT analysis, Dragonfly was used to reconstruct two-dimensional projections to provide axial cross-sections. A sub-volume composed of 300 z-stacked images was selected to evaluate the porosity, where the porosity of the PU@MXENE + CNT films was calculated according to the ratio of pore volume to solid volume. The porosities of Layer 2 of the four films were 67.84, 66.93, 67.09, and 67.47%, indicating that they were mostly consistent. Except for the 10%PU@MXene + CNT film, the porosities of Layer 1 were 62.1, 55.05, and 45.23%, indicating an obvious decreasing trend as the gradient increased. As shown in Figure 3e–h, the total void volume and interconnected void regions represented by specific colors obviously decreased with a reduction in porosity. The results indicated that the PU concentration influenced the porous structure of the film. The results in Supplementary Materials File also present the three-dimensional dynamic effects of the film.

Figure 3i–k displays the volume distributions of the films with different gradients. Among these, green represented Layer 1 in the gradient porous film, and orange represented Layer 2. The results showed that the volume of small pores with a pore volume of less than 2.5 μm^3^ accounted for most of the total pore volume. The volume distribution states of the pores in Layer 2, represented by orange in the four samples, were consistent. In addition, in the gradient porous film, within the range of small pores with a volume of less than 2.5 μm^3^, the pore volume of Layer 1 was greater than that of Layer 2. Within the range of large pores with a volume of greater than 2.5 μm^3^, the pore volume of Layer 1 was less than that of Layer 2. This was because the mass fraction of PU in Layer 1 in the gradient porous film was larger, resulting in a smaller formed pore volume.

**Figure 4:**
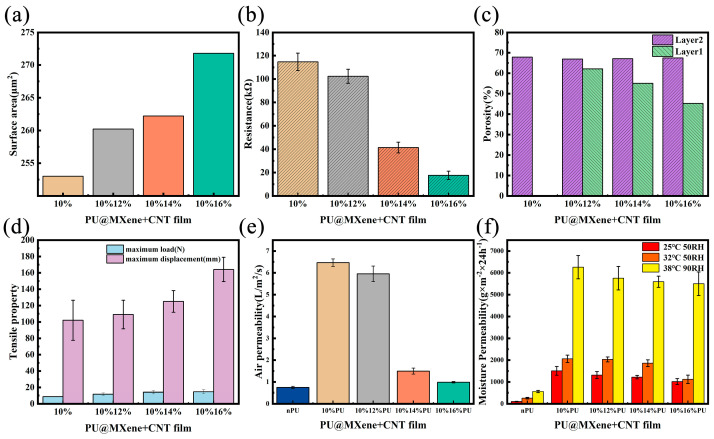
Properties of porous films with different gradients: (**a**) surface area, (**b**) surface resistance, (**c**) porosity, (**d**) tensile properties. Properties of porous films with different gradients and non-porous (nPU) films: (**e**) air permeability; (**f**) moisture permeability.

Figure 4a presents the surface area of the gradient porous films calculated by micro-CT, indicating that the specific surface areas of the gradient porous films were 253.1, 260.325, 262.305, and 271.845 μm^2^. As the degree of gradient porosity increased, the surface area of the film increased, with a greater number of MXenes and CNTs attached to the larger specific surface area, improving the conductivity of the film. As shown in Figure 4b, the resistance values of the four gradient porous films were 114.99 ± 7.40, 34.84 ± 5.90, 21.83 ± 4.67, and 14.64 ± 3.83 KΩ, and the resistance of the films obviously decreased. Due to the larger specific surface area, the resistance of the 10%16%PU@MXene + CNT film was 12.3% that of the 10%PU@MXene + CNT film. Therefore, the degree of gradient porosity affected the surface area and the resistance of the film due to the different pore structures. As shown in Figure 4c, the porosities of Layer 2 of the four gradient porous films were nearly consistent, at 67.84, 66.93, 67.09, and 67.47%, and the porosities of Layer 1 were 62.1, 55.05, and 45.23%, indicating an obvious decreasing trend. Figure 4d illustrates the tensile resistance of the four gradient porous films. As the degree of gradient increased, the tensile stress and maximum tensile displacement also gradually increased, increasing from 8.67 N and 102.07 mm for the 10% PU@MXene + CNT film to 14.70 N and 164.14 mm for the 10%16% PU@MXene + CNT film. This could be attributed to the fact that when the degree of the gradient of the composite film was low, larger pores formed, the porosity was higher, and Young’s modulus of the porous film was lower, which was manifested as a weaker ability to resist tension and lower tensile stress. When the composite film exhibited a high gradient degree, it demonstrated improved tensile resistance due to the opposing effect.

The air and water permeability serves as a key property for achieving the long-term comfort of wearable sensors. Figure 4a–d shows that due to its internal porous structure, the PU@MXene + CNT porous film exhibited high air permeability, enabling the effective penetration of air and water vapor. Figure 4e presents the air permeability of the porous film, which was compared to that of commonly used non-porous films (nPU). The results showed that the porous film had higher air permeability, with a maximum value of 6.4988 L/m^2^/s, and the air permeability values of the four porous films were 8.81, 8.11, 2.04, and 1.35 times that of the non-porous film. Figure 4f presents the moisture permeability of the porous film, which was compared with that of the commonly used non-porous film (nPU). The results showed that the porous film had higher moisture permeability. Under three different temperature and humidity conditions, the maximum values reached 1509.6, 2060, and 6258.8 g/m^2^/day. Under optimal conditions for human comfort, requiring 32 °C and 50% RH, the evaporation rate of the skin surface was 1900 g/m^2^/day. At this point, the moisture permeabilities of the porous films were 2060, 2034.8, 1860, and 1123.6 g/m^2^/day, with values 8.05, 7.96, 7.28, and 4.40 times that of the non-porous PU film, respectively, thereby essentially meeting the comfort requirements of human skin. Therefore, the air and moisture permeabilities of the porous film decreased with reduced porosity; however, the gradient porous film with the lowest air permeability was still significantly higher than the non-porous film.

**Figure 5:**
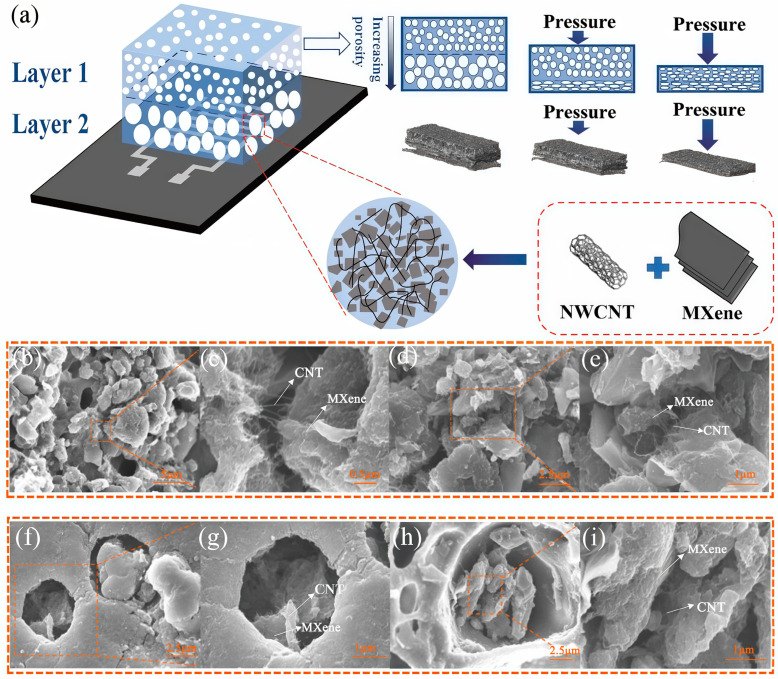
(**a**) Schematic diagram showing the compression process of the gradient porous film; (**b**–**e**) distribution of MXenes and CNTs on the surface of the gradient porous film and their magnified views; (**f**–**i**) distribution of MXenes and CNTs inside the gradient porous film and their magnified views.

The pressure sensing capabilities of the gradient porous PU@MXene + CNT sensor are shown in Figure 5a. When a film with a uniform porous structure was subjected to external pressure, it experienced compressive deformation along the entire thickness direction. When the pore walls came into contact, the conductive paths changed, resulting in a significant change in resistance. However, under a large pressure range, the pore walls of the film were already in contact and squeezed. At this point, the resulting change in resistance was small, and the sensitivity of the sensor under large pressure was low. As shown in Figure 5a, the film with a gradient porous structure was different from films with a uniform porous structure. When external pressure was applied, due to the difference in Young’s modulus of the gradient porous materials, the film with larger pores had a lower Young’s modulus, while the film with smaller pores had a higher Young’s modulus. Therefore, Layer 2 in gradient porous film was compressed first due to its higher porosity. When the pore walls came into contact, a large change in resistance occurred, and at this time, it exhibited higher sensitivity. When the pressure was further increased, the film with larger pores had already been squeezed together, resulting in only a small range of resistance changes when the pressure was increased. In contrast, the film with smaller pores can be further compressed, leading to a noticeable continuous change in resistance. Layer 1, with a lower porosity, became the main stress-bearing layer. At this time, the pore walls in Layer 1 came into contact, and a second large change in resistance occurred. When incorporated into a flexible sensor, the material maintained high sensitivity across a wide detection range.

The conductive network consisted of one-dimensional tubular CNTs and two-dimensional sheet-like MXenes. As shown in Figure 5b–e, these conductive materials were uniformly distributed on the surface of the porous structure, and Figure 5f–i illustrates their uniform attachment within the internal pores. This complex arrangement of conductive pathways enhanced the sensor’s performance. A hierarchical conductive network is formed by the interconnection of two-dimensional MXene with large MXene flakes, the small-area connection between one-dimensional CNTs, and the bridging of the pores between adjacent large MXene flakes, as well as the pores within the layers of the multilayer MXene by smaller-volume CNTs. This network endows the gradient porous structure sensing layer with more significant resistance changes than single-dimensional conductive materials. Within the low-voltage range, the contact between the relatively large MXene flakes and the CNTs on their surface mainly determines the change in resistance. As the pressure range gradually increases, the further contact of one-dimensional CNTs with inconsistent orientation distributions, the contact between the layers of the multilayer MXene itself, and the contact of CNTs distributed between the layers of MXene itself mainly account for the resistance change. This enables the sensing layer to exhibit a significant resistance change rate even within a relatively large pressure range.

**Figure 6:**
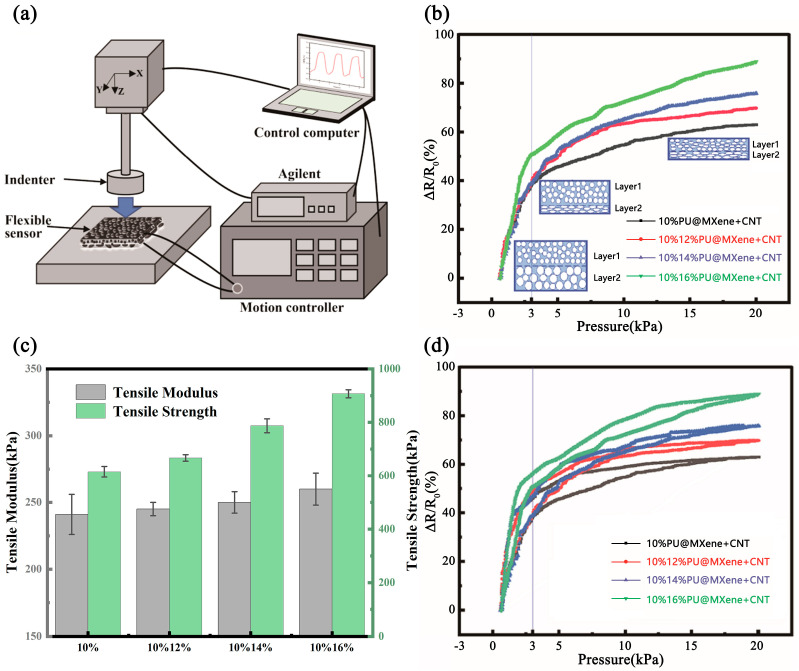
Sensing performance of different gradient porous PU@MXene + CNT sensors: (**a**) schematic diagram of the device for testing the sensing performance; (**b**) resistance change with increasing pressure; (**c**) tensile modulus and tensile strength; (**d**) cyclic pressure resistivity response.

As shown in Figure 6b, when a uniform pressure was applied between 0 and 20 kPa, the relative resistivity changes of all sensors gradually increased. Sensitivity can be defined as follows:

(1) $$S=\delta (\Delta R/R_{0})/\delta P,$$

(2) $$\begin{array}{l} \Delta R=R*R_{0} \end{array},$$

where Δ*R*, *R*_0_, and *P* represent the resistance, resistance before applying pressure, and change in applied pressure, respectively. The sensing range could be divided into two regions: 0.7~3 kPa and 3~20 kPa.

In the pressure range of 0.7~3 kPa, the sensitivities of the 10% PU@MXENE + CNT film, 10%12% PU@MXENE + CNT film, 10%14% PU@MXENE + CNT film and 10%16% PU@MXENE + CNT film are 22.663486 kPa^−^^1^, 22.67116 kPa^−^^1^, 22.854632 kPa^−^^1^, and 31.7912 kPa^−^^1^, respectively. The sensor with a gradient degree of 6 exhibits the highest sensitivity, which can be attributed to the inconsistent pore morphology between the upper and lower layers. Figure 3 shows that in the gradient porous membrane, within the range of small pores smaller than 2.5 μm^3^, Layer 1 > Layer 2; within the range of large pores larger than 2.5 μm^3^, Layer 1 < Layer 2. Since both the upper and lower layers are made of polyurethane material, and the pores of Layer 2 are larger in both pore volume and pore diameter than those of Layer 1, Young’s modulus of Layer 2 is lower. Therefore, Layer 2 becomes the main working layer at this stage and is compressed first. The 10%16% PU@CNT membrane exhibits the most pronounced overall hierarchical structure. Upon exposure to external stress, the working layer of the 10%16% PU@CNT film composite membrane is concentrated in Layer 2 to the greatest extent. Consequently, under the same external force, it shows the most significant change in pore volume and the highest resistance change rate, surpassing those of the 10%PU@MXene + CNT film, 10%12% PU@MXene + CNT film, and 10%14% PU@MXene + CNT film composite membranes.

In the pressure range of 3~20 kPa, the sensitivities of the 10% PU@MXENE + CNT film, 10%12% PU@MXENE + CNT film, 10%14% PU@MXENE + CNT film and 10%16% PU@MXENE + CNT film are 1.64 kPa^−1^, 1.27 kPa^−1^, 1.66 kPa^−1^, and 2.06 kPa^−1^, respectively. The sensitivities of the four samples all showed a decrease to varying degrees. When the pressure increases, the pores throughout the thickness of the membrane are further squeezed and compressed, causing a change in the resistance of the single-layer porous structure. This results in a significant decrease in the relative change rate of resistance compared to the process where the pore walls change from being initially separate to making contact within the 0.7~3 kPa range. In the gradient porous membrane, within the range of small pores smaller than 2.5 μm^3^, Layer 1 > Layer 2. Since both the upper and lower layers are made of polyurethane material, Young’s modulus of the porous structure of Layer 1 is higher than that of Layer 2. Consequently, the main working layer of the hierarchical porous structure shifts to Layer 1 at this stage. Given that Layer 1 is more resistant to compression compared to Layer 2, a certain degree of sensitivity reduction occurs. There is still a difference in sensitivity, and it is clearly shown that the greater the degree of hierarchical porosity, the greater the sensitivity. At this time, the main working layer becomes Layer 1. Under relatively high pressure, the hierarchical pores with a lower degree of porosity have already been squeezed together, resulting in only a small range of resistance change when the pressure is increased. In contrast, those with a higher degree of hierarchical porosity can be further compressed, leading to a noticeable continuous change in resistance. The 10%16%PU@MXene + CNT film stands out due to the largest number of small pores (volume < 2.5 μm^3^) in Layer 1 among the samples, endowing it with the highest Young’s modulus for Layer 1. While the pores in other samples become fully compressed and lose the ability to deform significantly under load, the 10%16%PU@MXene + CNT film retains the capacity for substantial deformation, leading to the most significant change in the relative resistance rate and, consequently, exhibiting the highest sensitivity.

As shown in Table 1, the 10%16%PU@MXENE + CNT film has the highest sensitivity, which is 31.7912 kPa^−^^1^, and its repeatability is 8000 cycles, significantly higher than recently reported sensors based on surface microstructures.

Figure 6d shows the cyclic piezoresistive curve. To quantify the correlation of the porous PU@MXENE + CNT, the hysteresis error could be defined as follows:

(3) $$\begin{array}{l} H=\pm \left(\frac{\Delta H_{max}}{YFS}\right)*100\% \end{array},$$

(4) $$\begin{array}{l} \begin{array}{l} \Delta H=\left|RL(s,t)Ru(s,t)\right| \end{array} \end{array}$$

where Δ*H*_max_ represents the maximum deviation between one cycle and another and could be derived as the maximum value of the full-scale output. As in Figure 6d, 10% PU@MXENE + CNT had a larger hysteresis window due to its larger pore structure, with a hysteresis error of ±13.4%. The hysteresis errors of 10%/12% PU@MXENE + CNT, 10%/14%PU@MXENE + CNT, and 10%/16%PU@MXENE + CNT were ±9.8%, ±8.2%, and ±7.1%, respectively. Compared to 10%PU@MXENE + CNT, the hysteresis errors were optimized by 26.5, 39.0, and 46.7%. As shown in Figure 6c, the tensile moduli of the 10%PU@MXENE + CNT, 10%12%PU@MXENE + CNT, 10%14%PU@MXENE + CNT, and 10%16%PU@MXENE + CNT films are 241.67 kPa, 245.49 kPa, 252.28 kPa, and 260.15 kPa, respectively. Their corresponding tensile strengths are 615.23 kPa, 667.37 kPa, 787.19 kPa, and 905.58 kPa, respectively. The tensile modulus and tensile strength of the film increase with the increase in the degree of gradient grading between Layer 1 and Layer 2. Pores with larger volumes formed at a low degree of gradient grading exhibit weak tensile resistance. In contrast, a high degree of gradient grading leads to an increase in small pores with smaller volumes and uniform pore sizes, endowing the material with enhanced tensile resistance. In contrast to a structure featuring solely large pores, the gradient porous structure exhibits a gradient variation in pore size. Upon application of force, the regions with smaller pores initially bear the stress and subsequently transfer it step-by-step to the regions with larger pores. This mechanism effectively prevents stress concentration around the pores, enabling more harmonious deformation within the material. It reduces the hysteresis phenomenon. Experiments demonstrated that a gradient porous structure could effectively reduce the hysteresis phenomenon and errors in wearable sensors.

**Figure 7:**
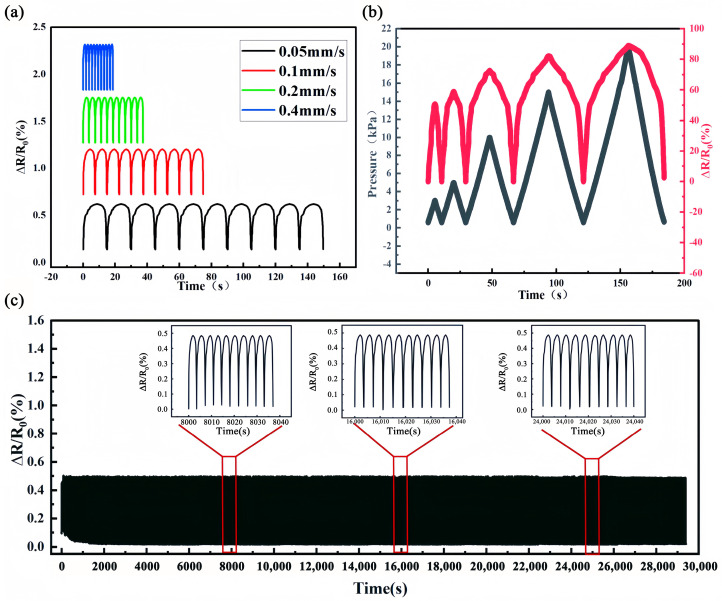
(**a**) Change in resistance change rate over time at different compression speeds; (**b**) change in resistance change rate over time under different loads; (**c**) change in resistance change rate of 10%/16%PU@MXene + CNT over time under 8000 loading cycles.

Figure 7a presents the change in resistance change rate over time when loading and unloading pressures with different application rates were applied in the range of 0–3 kPa. Figure 7b presents the variation range of different resistance change rates of 10%/16%PU@MXene + CNT under the corresponding applied pressures, implying that the pressure sensor could respond sensitively to external stimuli. Figure 7b shows the stability of 16%PU@MXene + CNT after 8000 cycles in the range of 0–3 kPa, and as shown in Figure 7c, 10 cycles were selected from cycles 2000, 4000, and 6000 for analysis. Therefore, the results indicated that the porous PU@MXENE + CNT piezoresistive sensor had good repeatability and stability.

**Figure 8:**
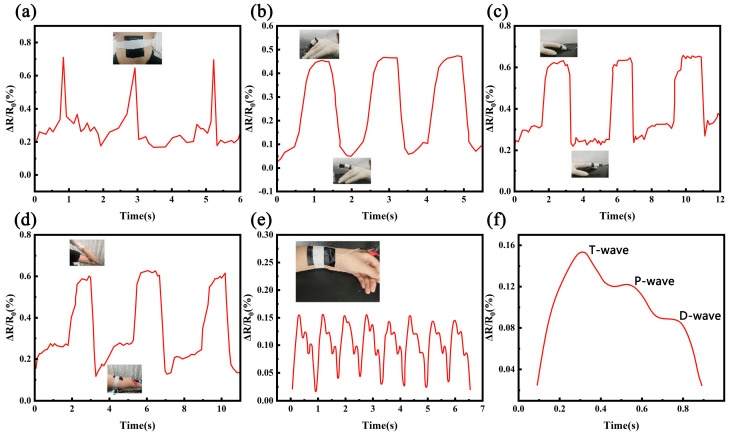
Real-time detection using a flexible piezoresistive sensor based on the gradient porous structure of PU@MXene + CNT; (**a**) throat swallowing action detection; (**b**) finger bending action detection; (**c**) detecting a pulse at the wrist in the relaxed state; (**d**) arm bending action detection; (**e**) finger pressing action detection; (**f**) magnified section of (**c**).

Due to its superior performance, we performed real-time monitoring using the porous PU@MXENE + CNT sensor in this work. With the assistance of medical tape, this breathable and wearable sensor can be comfortably attached to the uneven surface of human skin. When a person drank water, the movement around the throat produced a special resistance response. As shown in the figures, the resistance response could be recorded, and the same waveform could be stably obtained, as shown in Figure 8a. Figure 8b,c shows that the sensor attached to the finger could be used to detect bending and pressing. When the index finger was repeatedly bent and relaxed, the resistance changed by about 45%. In the case of finger pressing, the resistance change was equivalent to 60%, which was significantly higher than that when the index finger was repeatedly bent and relaxed. Figure 8d shows the detection of arm bending using this sensor, while Figure 8e,f shows the atrial pulse signals of volunteers monitored by the porous PU@MXENE + CNT sensor. As shown in the figure, the pulse rate of the volunteer in a relaxed state was 74 beats/min. Specifically, the sensor could distinguish the detailed characteristic peaks of a typical human pulse waveform, which was associated with “P” (percussion wave), “T” (tidal wave), and “D” (diastolic wave).

According to the above results, the gradient porous PU@MXENE + CNT sensor with good air and moisture permeability could sensitively distinguish different pressure changes, providing unlimited potential for its use as electronic skin.

## 4. Conclusions

This study introduced a wearable piezoresistive sensor with high sensitivity, a wide detection range, and good air and moisture permeability. Different gradient porous PU@MXene + CNT films were prepared by controlling the PU concentration. SEM confirmed good interfacial interaction between MXenes, CNTs, and PU, and the structure of the PU@MXene + CNT film was reconstructed using micro-CT. According to the micro-CT analysis results, the PU concentration directly affected the pore morphology, and the PU porous structure with the largest difference in mass fraction had the highest degree of gradient porosity. The PU@MXene + CNT porous film had good air and moisture permeability. Specifically, the air permeability of the 10% PU@CNT film was 881% that of the non-porous PU film, and the moisture permeability of the PU@MXene + CNT film was 805% that of the non-porous PU film. Meanwhile, as the specific surface area of the PU@MXene + CNT porous film increased, corresponding to an increase in the degree of gradient, the resistance of the PU@MXene + CNT porous film decreased. In addition, based on the porous structure of the PU@MXene + CNT film, the assembled wearable sensor exhibited excellent piezoresistive performance. Under a pressure of 0.7–3 kPa, the sensitivity of the porous PU@CNT sensor reached a maximum of 31.79 kPa^−1^. In the 3–20 kPa compression range, the sensitivity of the porous film with the highest gradient was 2.06 kPa^−1^, which was still 20.07% higher than that of the non-gradient porous film. Finally, to ensure its ability to be used as electronic skin, the porous PU@MXene + CNT sensor was used to detect movements such as throat movement during swallowing, finger pressing and bending, and arm bending. The sensor was also tested for detecting pulse pressure signals at the wrist, where it exhibited reliable performance. With its combined properties, the porous PU@MXene + CNT sensor offers strong potential for various applications in wearable electronic devices.

## Figures and Tables

**Table 1:** Comparison of the main performance of the sensor with other recently reported sensors based on surface microstructures.

Ref.	Sensitivity (kPa^−1^)	Sensing Range (kPa)	Durability (Cycles)
[7]	1.16	1500	1000
[10]	3.64	0–20	2000
[11]	2.57	0.25–2	225
[20]	0.48	0–5	2000
[32]	1.17	0–47	--
This work	31.7912	0–20	8000

## Data Availability

The original contributions presented in this study are included in the article/Supplementary Material. Further inquiries can be directed to the corresponding authors.

## Supplementary Materials

The following supporting information can be downloaded at: https://www.mdpi.com/article/10.3390/polym17111530/s1, Figure S1: (**a**) The change in electrode resistance over time under 300 bending cycles. (**b**) Pictures of the electrode after the cycling test; Figure S2: Tensile properties of 10%16% PU and 10%16% PU@MXene + CNT.
