# Supplementary material for: A Breathable, Highly Sensitive, and Wearable Piezoresistive Sensor with a Wide Detection Range Based on Gradient Porous PU@MXene/CNT Film for Electronic Skin
Source: Polymers (Basel). 2025 May 30;17(11):1530. doi: 10.3390/polym17111530 (PMC12158107; doi:10.3390/polym17111530)
Supplement: Supplementary file 1 [file polymers-17-01530-s001.zip › polymers-3630374-supplementary.pdf]

polymers

MDPI

Article

A Breathable, Highly Sensitive, and Wearable Piezoresistive Sensor with a Wide Detection Range Based on Gradient Porous PU@MXene/CNT Film for Electronic Skin

Xiuli Yang [1], Feiran He [1], Huihui Qiao [1], Shuibo Yang [1], Dehua Wen [1], Kaige Yang [1], Ziyi Dang [1] and Yin He [1,2,3,4,*]

1 School of Textile Science and Engineering, Tiangong University, Tianjin 300387, China; 13011335551@126.com (X.Y.); 15064768326@163.com (F.H.); 18222477692@163.com (H.Q.); 14777537638@163.com (S.Y.); 13211079562@163.com (D.W.); yangkaige2022@163.com (K.Y.); 13772715657@163.com (Z.D.)
2 Shaoxing Keqiao Institute, Tiangong University, Shaoxing 312030, China
3 Institute of Intelligent Wearable Electronic Textiles, Tiangong University, Tianjin 300387, China
4 Ministry of Education Key Laboratory for Advanced Textile Composite Materials, Tiangong University, Tianjin 300387, China
* Correspondence: heyin@tiangong.edu.cn

Supporting

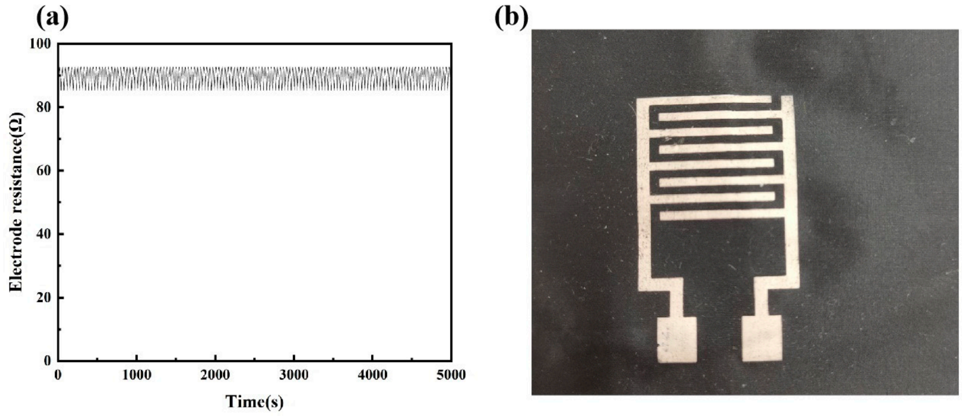

Figure S1. (a)The change in electrode resistance over time under 300 bending cycles. (b) Pictures of the electrode after the cycling test**.**

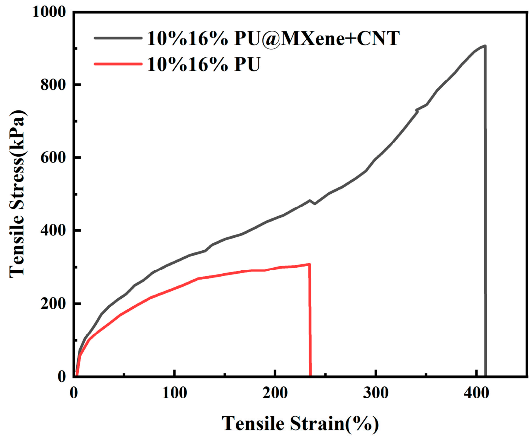

Figure S2. Tensile properties of 10%16% PU and 10%16% PU@MXene+CNT.
